# γ-H2AX as a Marker for Dose Deposition in the Brain of Wistar Rats after Synchrotron Microbeam Radiation

**DOI:** 10.1371/journal.pone.0119924

**Published:** 2015-03-23

**Authors:** Cristian Fernandez-Palomo, Carmel Mothersill, Elke Bräuer-Krisch, Jean Laissue, Colin Seymour, Elisabeth Schültke

**Affiliations:** 1 Stereotactic Neurosurgery and Laboratory for Molecular Neurosurgery, Freiburg University Medical Center, Freiburg, Germany; 2 Medical Physics and Applied Radiation Sciences Department, McMaster University, Hamilton, Ontario, Canada; 3 European Synchrotron Radiation Facility, Grenoble, France; 4 Institute of Pathology, University of Bern, Bern, Switzerland; 5 Department of Radiotherapy/Laboratory of Radiobiology, Rostock University Medical Center, Rostock, Germany; National Cheng Kung University, TAIWAN

## Abstract

**Objective:**

Synchrotron radiation has shown high therapeutic potential in small animal models of malignant brain tumours. However, more studies are needed to understand the radiobiological effects caused by the delivery of high doses of spatially fractionated x-rays in tissue. The purpose of this study was to explore the use of the γ-H2AX antibody as a marker for dose deposition in the brain of rats after synchrotron microbeam radiation therapy (MRT).

**Methods:**

Normal and tumour-bearing Wistar rats were exposed to 35, 70 or 350 Gy of MRT to their right cerebral hemisphere. The brains were extracted either at 4 or 8 hours after irradiation and immediately placed in formalin. Sections of paraffin-embedded tissue were incubated with anti γ-H2AX primary antibody.

**Results:**

While the presence of the C6 glioma does not seem to modulate the formation of γ-H2AX in normal tissue, the irradiation dose and the recovery versus time are the most important factors affecting the development of γ-H2AX foci. Our results also suggest that doses of 350 Gy can trigger the release of bystander signals that significantly amplify the DNA damage caused by radiation and that the γ-H2AX biomarker does not only represent DNA damage produced by radiation, but also damage caused by bystander effects.

**Conclusion:**

In conclusion, we suggest that the γ-H2AX foci should be used as biomarker for targeted and non-targeted DNA damage after synchrotron radiation rather than a tool to measure the actual physical doses.

## Introduction

Conventional radiotherapy has been very successful at treating a wide variety of cancers such as those of skin and breast, but it still remains poorly effective when targeting malignant brain tumours. Brain tumours account for 2% of all cancer in adults and represent the second cause of deaths in children after leukemia [1]. Unfortunately, the most frequent tumours—glioblastoma multiforme (GBM) in adults, and astrocytic tumours in children—are both the most aggressive and resistant to radiation therapy [2,3]. New therapy are required to improve prognosis.

Synchrotron radiation is a promising approach for brain radiotherapy. Research on microbeam radiation therapy (MRT) in the last two decades, initiated at the National Synchrotron Light Source (NSLS) at Brookhaven National Laboratory (BNL), Upton, New York, was continued at the European Synchrotron Radiation Facility (ESRF) and at other international facilities. Results of this research have repeatedly and consistently shown, in the hands of many different teams of scientists, that single-fraction MRT yields a larger therapeutic index than does a single irradiation by a single broad beam, for aggressive tumours such as transplanted intracerebral rat 9L gliosarcomas or F98 tumours [4–6]; for transplanted subcutaneous murine mammary carcinomas [7]; for the aggressive and invasive, extraordinarily radioresistant murine squamous cell carcinoma VII [8], and for other tumours. The remarkable sparing by x-ray microbeams of normal tissues of vertebrates—particularly of the normal brain and spinal cord—has been extensively documented in suckling and adult rats [5,9–15], duck embryos [16], and weanling piglets [17]. The skin also tolerates relatively high doses of x-rays delivered by microbeams [18,19]. Further, MRT-associated bystander effects have been identified [20–22], and gene expression analysis of intracerebral gliosarcomas in rats have identified MRT-induced immune modulations [23] and cytostatic effects [24].

Among the potential advantages of MRT over temporally fractionated, conventional radiotherapy, we note 1) the very short time required for treatment, i.e., 1–2 days for MRT rather than several weeks; animals positioned online in the MRT hutch of ID 17 at the ESRF can be imaged just before irradiation, and changing from imaging to irradiation takes less than a minute [25]; 2) the normal organ tolerance particularly of the normal brain and spinal cord, which might allow re-irradiation; 3) the ability to treat tumours with radiobiologically higher doses, with possibly improved local control rates. Radiation oncologists familiar with MRT deem that it might enable the palliation of central nervous system malignancies in infants and young children who at present cannot be safely and effectively palliated by existing radiotherapies or by any other kinds of therapies (for instance, diffuse pontine gliomas).

From a technical point of view, synchrotron radiation is typically used as an array of microbeams. The high photon flux of synchrotron-generated x-rays is spatially fractionated by the insertion of a collimator, which produces an array of quasi-parallel microbeams with intermediate gaps [26]. This particular microbeam configuration exposes areas of tissue to either peak or valley doses. The former corresponds to the tissue regions where the primary dose is deposited by the photons of the microbeam, while the latter refers to the secondary dose deposited in the tissue regions between the microbeams by the scattered photons [27]. The valley dose varies between approximately 2% to 10% of the peak dose, depending on the configuration and size of the beam array [4]. Microbeam widths typically vary from 20 to 100μm, with gaps of 200–400 μm wide. Having gap widths of 4 to 8 times greater than the width of microbeams, the volume of tissue affected by the valley dose would be 4^3^–8^3^ times larger than the tissue volume targeted by the peak dose. The ratio between the dose of the microbeams and the dose of the gaps is called peak-to-valley dose ratio (PVDR), which plays a major role in decreasing the dose to normal tissue. Thus PVDR is an important parameter in understanding the tissue tolerance to the high doses used in MRT [26].

There are several hypotheses concerning the biological basis of the tumouricidal effect of microbeams: Intracerebral transplantable tumours of rats are killed because the “valley dose” (i.e. the radiation leakage between the microbeams produced by Compton scattering, particularly in the transitional zones sited between peaks and valleys) is very high. High valley doses, given in a single dose fraction, augmented by the “dose spikes” from the “peak doses” of the microbeams, might be high enough to destroy the tumour’s abnormal microvasculature, but too low to destroy the microvasculature of normal tissues [4]. This may be the case in small animals where the valley dose is approximately the same in the tumour and in the normal tissues proximally and distally to the tumour. Conversely, in deep tumours of large animals, the valley dose would be higher in the normal tissues proximal to the tumour than in the tumour because of a much higher incident dose has to be applied to compensate for greater x-ray attenuation [28,29]. Also, the tissue within the valley regions is of particular interest because, depending on the peak dose and the radiation geometry, the dose deposited can be as low as 0.5 Gy, which is relevant for the induction of bystander effects [20].

Radiation-induced bystander effects (RIBE) are described as the extent of damage in cells that were not exposed to direct irradiation, but that after receiving signals from irradiated cells respond similarly as if they had been irradiated [30,31]. RIBE are relevant for MRT because 1) the tissue in the dose valleys may respond to signals released by the cells in the path of the microbeam [20], and 2) the tissue in the dose valleys will also receive low doses of radiation (i. e. scatter) that may allow the cells to produce bystander signals to then induce bystander effects on distant organs. Studies trying to identify the bystander molecule have found the involvement of extracellular mediators and intracellular pathways. Within the former we can identify reactive oxygen species (ROS) [32,33], reactive nitrogen species (RNS) [34], interleukin-8 (IL8) [35], tumour necrosis factor-α (TNF-α) [36], transforming growth factor-β1 (TGF-β1) [36], serotonin [37,38] and exosomes as the latest candidate [39,40]. Within the latter we find mitogen-activated protein kinases (MAPKs), the NF-κB transcription factor, COX2, NOS2 [36,41], mitochondrial disruptions [42–44], Ca^2+^ fluxes [45], and expression of apoptotic and cell cycle regulatory molecules like p53, p21^Waf1^, p34, and MDM2 [46–48]. Moreover, the latest research show that another candidate for bystander triggering factor is UV-photon emission from irradiated cells [49,50].

Our group has previously explored the occurrence of bystander effects in tissue from irradiated and non-irradiated rat brains [21]. The results of that study suggested that the yield of bystander signals was higher in Wistar rats harbouring C6 gliomas than in tumour-free rats. Moreover, protein formation after synchrotron radiation has also been explored, showing that the bystander-induced proteome may confer anti-tumorigenic properties that are based on ROS-induced apoptosis [22]. The probability of bystander signals being communicated between animals was also investigated. Wistar rats received synchrotron radiation to their right cerebral hemisphere and were then paired with unexposed rats over 48 hours [51]. The results showed that radiation damage was effectively communicated between animals through bystander signals.

A major challenge with synchrotron radiation is tracking and quantifying the dose deposition in the tumour and in normal tissue. One of the most reliable markers for DNA damage after radiotherapy is γ-H2AX. This molecule describes the phosphorylation of the H2AX histone on serine 139 [52]. γ-H2AX was first demonstrated to appear as rapidly as 1 min after ionizing radiation exposure, reaching its maximum formation at 10 min [52]. The authors concluded that γ-H2AX was directly related with double strand breaks (DSBs) at a formation rate of 1% per gray. However, recent studies have challenged that view. Work published by Costes et. al. shows that γ-H2AX is a DNA damage sensing protein that does not necessarily correlate with DSBs. Instead, it may operate as a rigid scaffold on regions of chromatin to keep broken DNA in place while allowing DNA repair enzymes to access the damaged site [53]. γ-H2AX has also been evaluated as a biomarker to predict radiation sensitivity. Greve et al. used the γ-H2AX marker to predict the clinical radiosensitivity of patients after cancer treatment [54]. Although they observed that peripheral blood lymphocytes extracted from patients irradiated with 2 Gy produced a maximum of H2AX phosphorylation 1 hour after irradiation, no satisfactory conclusion about radiation sensitivity could be made. Nevertheless, these studies agreed that γ-H2AX formation is a rapid and sensitive cellular response to radiation stress, which makes it an important marker of dose deposition.

The use of γ-H2AX after synchrotron radiation has been explored in monolayers of cells [55], the skin of healthy mice [7] and in mice harbouring skin tumours [7]. Our group started to look at the use of γ-H2AX in mouse brain after synchrotron pencilbeam irradiation, where we demonstrated a correlation between dose and the formation of γ-H2AX foci [56]. The aim of the present work was to study the dose deposition of synchrotron radiation in the brain and cerebellum of rats after micro- and broad beams using the γ-H2AX marker under several conditions.

## Materials and Methods

### Animal Model

Adult male Wistar rats were housed and cared for in a 12-hour light/dark cycle in a temperature-regulated animal facility at the European Synchrotron Radiation Facility (ESRF). All experiments were performed according to the guidelines of the French and German Councils on Animal Care. This study was approved by the Institutional Animal Care and Use Committees of both European participating institutions (Freiburg University Medical Center and ESRF, G10-87).

The C6 glioma cell line was selected for our studies because it shares a wide range of characteristics with the highly malignant human brain tumour glioblastoma multiforme (GBM) [57]. Once injected into the brain, C6 gliomas rapidly proliferate forming a solid malignant tumour (morphologically similar to GBM), delineated by a rim of active astrocytes, with small groups of tumour cells migrating along the blood vessels [58]. C6 gliomas were originally produced as a result of exposing Wistar-furth rats to N-nitrosomethylurea, and then isolated and grown as a cell culture [59]. This tumour model has been used in multiple studies involving conventional radiotherapy [60–62] and synchrotron radiation [63–65].

For these experiments, C6 cells were obtained from the American Type Culture Collection and maintained in T75 cm^2^ flasks using Dulbecco's Modified Eagle Medium (Gibco, France) supplemented with 10% FBS (Gibco, France) and 5ml Penicillin-Streptomycin (Gibco, France). Cells from a 90% confluent culture were detached by incubation with 20 ml of Hank’s Balanced Salt Solution (Gibco, France) without calcium and magnesium for 20 minutes at 37°C in an atmosphere of 5% CO_2_ in air. The cell suspension was centrifuged at 1000 rpm for 4 min, the pellet was re-suspended in 1ml of fresh growth medium and cells were counted using a haemocytometer.

Wistar rats were subjected to general anaesthesia (2–2.5% isofluorane in 2 L/min compressed air) and placed in a stereotactic frame. An incision of 1 to 1.5 cm length was made on the scalp following the sagittal midline. A burr hole was placed in the skull over the right hemisphere, 3 mm to the right from the sagittal midline and 3 mm posterior from the coronal suture. Then 100,000 C6 cells suspended in 10 μl were slowly injected into the brain 3 mm below the cortical surface over 4 minutes, using an automated syringe pump (KDS 320, GENEQ). Once the injection was finished and the needle removed, the hole was sealed with bone wax and the incision was closed. Rats were maintained for 7 days to allow tumour development.

### Irradiation

**Fig 1:**
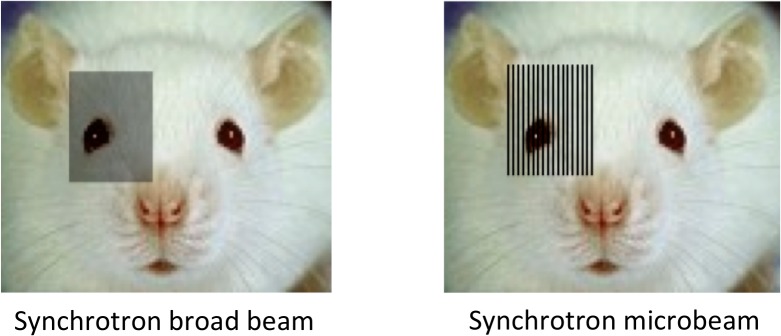
Schematic representation of a broad beam and a microbeam array.

Prior to irradiation, animals were deeply anesthetised using 3% isofluorane in 2L/min compressed air and an intraperitoneal injection of a Ketamine-Xylazine (61.5 /3.6 mg.kg^-1^ i.p.). Synchrotron radiation was delivered to the right cerebral hemisphere in anterior-posterior direction in a single session using either a broad beam or a microbeam configuration (Fig. 1). Broad beam refers to a synchrotron x-ray beam uniformly distributed within the irradiation field, resembling a conventional radiotherapy approach. Microbeam irradiation refers to a spatially fractionated synchrotron x-ray beam arranged in an array of alternating quasi-parallel microplanar beams and gaps. The broad beam was 14 mm high and 9.825 mm wide, and the doses delivered were 35, 70, or 350 Gy at the skin-entry level. The microbeam had a similar field size as the broad beam but it was composed of 50 alternating quasi-parallel rectangular microbeams of 25 μm width with a center-to-center distance of 200 μm. The peak doses delivered were the same as for broad beam irradiation: 35, 70 or 350 Gy at the skin-entry level. Gafchromic Films (HD-810 and MD-V2-55 films from Nuclear Associates, NY, USA) were used to verify all irradiation doses and modalities applied.

### Tissue Preparation

Rats were anesthetised and decapitated either at 4 or 8 hours after irradiation for the extraction of the brains, which were kept in 10% phosphate-buffered formalin overnight. The next morning, the brains were bisected into a superior and an inferior part in the horizontal plane. Both parts were placed in histology cassettes and immersed again in 10% phosphate-buffered formalin overnight. The brains were then embedded in paraffin and sectioned into 3 μm thick tissue sections, which were mounted on microscopy slides and stored at +4°C.

### Immunostaining

Tissue sections were deparaffinised and rehydrated through a series of alcohol and xylene washes. Vapour-based heat epitope retrieval was performed by putting the microscope slides in a citrate pH6 solution (Target retrieval solution, Dako, Germany, #S169984-2) at a 95°C for 40 minutes. The sections were then stained using the Shandon Sequenza Immunostaining Rack System. A coverplate was placed on the glass-slide to form a capillary gap that allows the staining reagents to flow through. The coverplate and slide assembly were placed in the rack and charged twice with PBS. Tissue sections were blocked with 100 μl of 1x PBS, 5% goat serum, and 0.3% triton X-100 buffer for 60 minutes at room temperature, followed by incubation with 1:100 dilution of γ-H2AX antibody (Abcam, Cambridge, UK) for 1 hour at room temperature. Slides were then rinsed 3 times with PBS and incubated with Alexa Fluor-488 (1:200 dilution), and DAPI for 1 hour at room temperature in the dark. Finally, slides were rinsed three times with PBS, the coverslips were placed using the Dako Fluorescent Mounting Medium and sealed with nail polish. Slides were stored flat at 4°C in the dark until image acquisition.

### Image acquisition and analysis

Images were acquired in the Laboratory of Molecular Neurosurgery in the Freiburg Neurocenter using an Olympus AX70 fluorescence microscope. Images were post processed using the Aperture 3.5.1 software, and analyzed at McMaster University.

Images were loaded into the Image Pro Plus 6.2.1 software, and three parameters were analyzed: 1) the width of the area covered by the γ-H2AX positive cells (radiation track) was measured in the cerebellum. 2) The intensity of fluorescence was measured through the use of intensity profile lines that were 100 μm long and traced perpendicular to the direction of the radiation tracks. The average values were then plotted as histograms using the Prism 6 software. 3) The number of γ-H2AX positive cells per micrometer-square was also detected. Images were analyzed by counting the number of γ-H2AX positive cells per micrometer area. A cell was counted as positive when it had strong fluorescence; cells with questionable positivity (weak or intermediate fluorescence) were defined as negative. The results were plotted accordingly using the Prism 6 software. Although a careful analysis of the results indicates that a comparison between the fluorescence intensities of peak and valley could enrich this study, it would require us to run the experiments again and we have no beam time to do this. For the aforementioned reason we highly recommend to any group running similar tests to consider a direct comparison between peak and valleys.

## Results

### γ-H2AX foci as a biomarker for dose deposition

**Fig 2:**
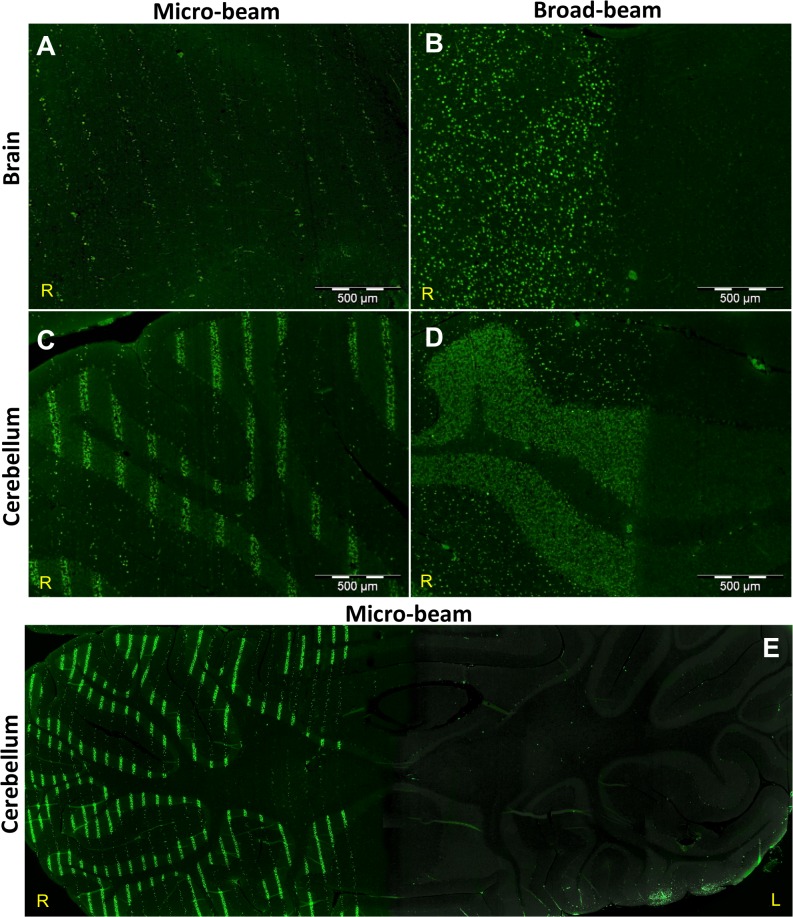
γ-H2AX stain comparison between micro- and broad beam configurations. Horizontal sections of the irradiated right cerebral hemisphere and the cerebellum of Wistar rats. Images A—D were obtained from the irradiated right cerebral hemisphere and cerebellum of animals exposed to 350 Gy of either microbeam or broad beam; dissected 8 hours after irradiation. For the MRT array, the center-to-center distance was 200 μm. The position and intensity of the γ-H2AX marker (green) correlate with the deposition of the peak synchrotron doses. A) Radiation tracks of the microbeams in the right cerebral hemisphere. B) Right cerebral hemisphere after broad beam irradiation C) Radiation tracks of the microbeams in the cerebellum. D) Cerebellum after broad beam irradiation. E) Horizontal section through the whole cerebellum after exposure of the right hemisphere to microbeams of 350 Gy; dissected at 4 hours after irradiation.

We analyzed the brain of Wistar rats after three different doses of synchrotron radiation (35, 75, and 350 Gy) using either microbeams or broad beams. The results demonstrated that the γ-H2AX antibody could effectively be used as a marker for dose deposition in the cerebral hemispheres and in the cerebellum. Brain images showing the exposure to 350 Gy of micro- and broad beams are shown in Fig. 2A and 2B respectively. The typical radiation tracks detected by the γ-H2AX antibody were observed in all brain samples exposed to synchrotron radiation. Because of the sharp dose gradient, the edges of the irradiation fields with both micro- and broad beams were well delineated. The cerebellum is shown in Fig. 2C and 2D. The higher cellular density of the granular cell layer allows a good visualization of the radiation tracks. Conversely, the cerebral hemispheres (2A & 2B) contain a lower amount of cell bodies and thus comparatively less DNA to be targeted. To complement the analysis, Fig. 2E shows the whole cerebellum after a microbeam exposure of 350 Gy. This image demonstrates the accuracy and precision of the synchrotron microbeam radiation technique. Due to an artefact of tissue distortion that occurs during the histological processing of the images, the radiation tracks do not always appear as perfectly straight lines.

**Fig 3:**
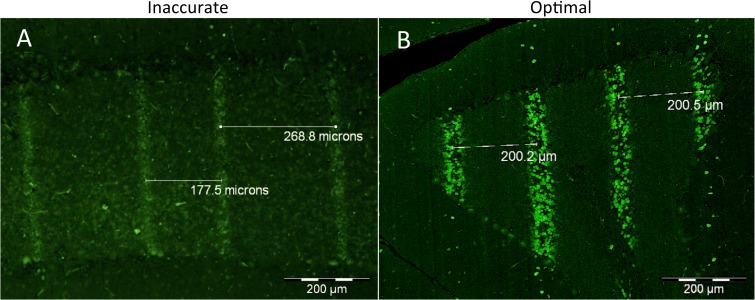
Comparison of radiation tracks produced by the same intended microbeam configuration (center-to-center distance of 200 μm). By design the collimator spatially fractionates the microbeams with a center-to-center distance of 400 μm; to generate a center-to-center distance of 200 μm the collimator moves laterally followed by a second passage of the animal through the beam. A) Variable center-to-center distance of the microbeams due to inaccurate lateral translation of the collimator (35 Gy, 4 hours after irradiation). B) Accurate delivery of the microbeams. (350 Gy, 8 hours after irradiation).

The comparison of γ-H2AX staining in 2 animals of the same cohort shown in Fig. 3 demonstrates the reliability of this stain in detecting unintended irradiation patterns due to technical problems during the irradiation process. In Fig. 3A, problems with the lateral translation of the collimator resulted in unequal spacing of the microbeams within the irradiation field in one of the animals, while Fig. 3B shows the correct delivery of the intended radiation pattern. This example indicates that the γ-H2AX marker can also be used to verify the accuracy of the delivery of the microbeams in situ.

### Factors that influence the formation of γ-H2AX foci

**Fig 4:**
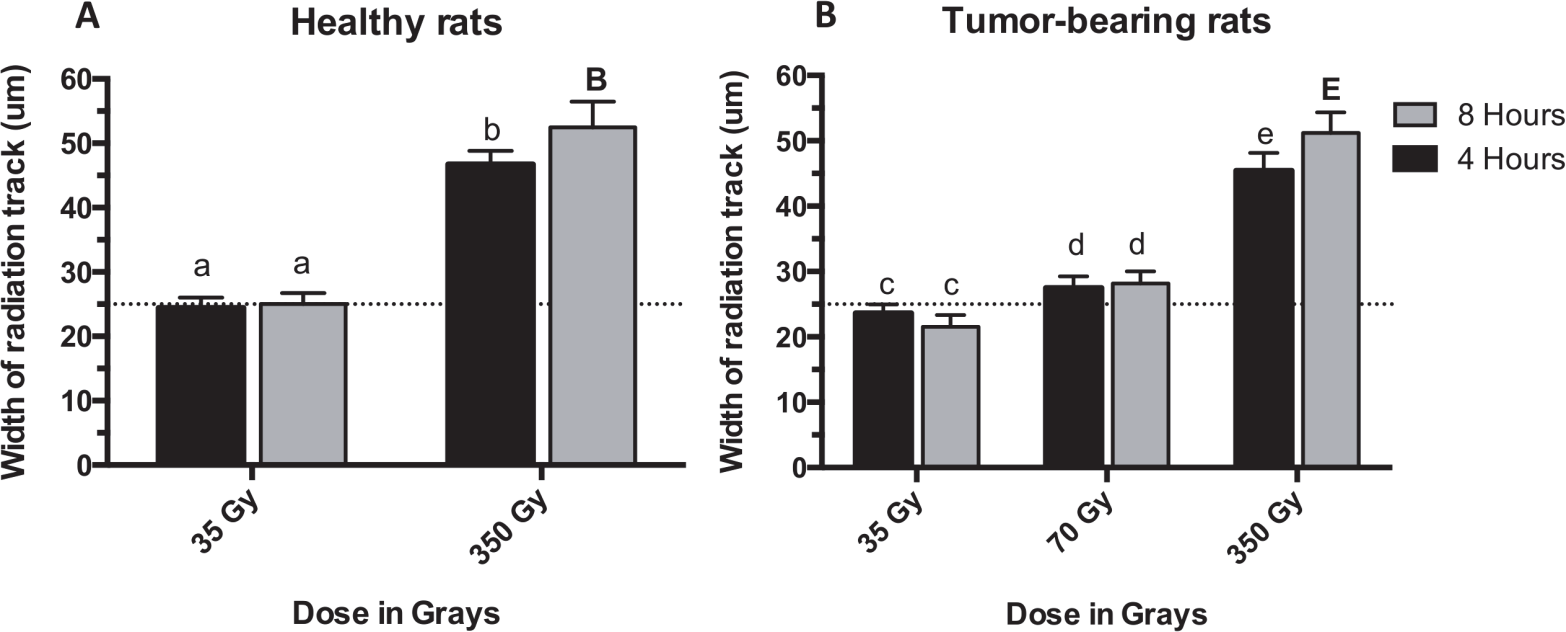
Mean thickness of the radiation tracks in the cerebellum. Microbeam irradiation was given to both normal (A) and tumour-bearing rats (B). The width of the microbeams was 25 μm, which is represented by the dotted line. Animals were exposed to 35, 70, or 350 Gy to their right cerebral hemisphere. Four and 8 hours indicate the two dissection times after irradiation. Different letters and different letter cases indicate significant differences between groups and within each group respectively. Error bars show SD.

Fig. 4 shows the width of the radiation path in micrometers, formed in the cerebellum by the phosphorylation of the H2AX histone to serine 139. Fig. 4A shows how the dose significantly modifies the width of the tracks in normal rats. The plotted values indicate that 1) 35 Gy produced radiation tracks that match the width of the original microbeam and 2) 350 Gy increased the width of the tracks almost twice the width of the original microbeam. Moreover, the width of the radiation track significantly increased (P value < 0.01) from 4 to 8 hours after 350 Gy, while that phenomenon did not occur after 35 Gy (P value = 0.9265).

Fig. 4B shows that in tumour-bearing rats the irradiation dose affected the width of the radiation tracks similarly as in normal rats. While 35 Gy produced tracks of almost 25 μm width, 70 Gy resulted in slightly higher values producing tracks of 27 μm. The irradiation dose of 350 Gy produced once again an increase in the width of the microbeam tracks to almost twice the original width of the 25 μm microbeams. Furthermore, there was a statistically significant increase (P value < 0.01) in the width of the microbeam tracks between 4 and 8 hours after irradiation while no significance was found after 35 Gy (P value = 0.1563) and 70 Gy (P value = 0.9727). To summarize one can state that normal and tumour-bearing animals seem to have the same degree of response to both the irradiation dose and the time interval between irradiation and dissection.

**Fig 5:**
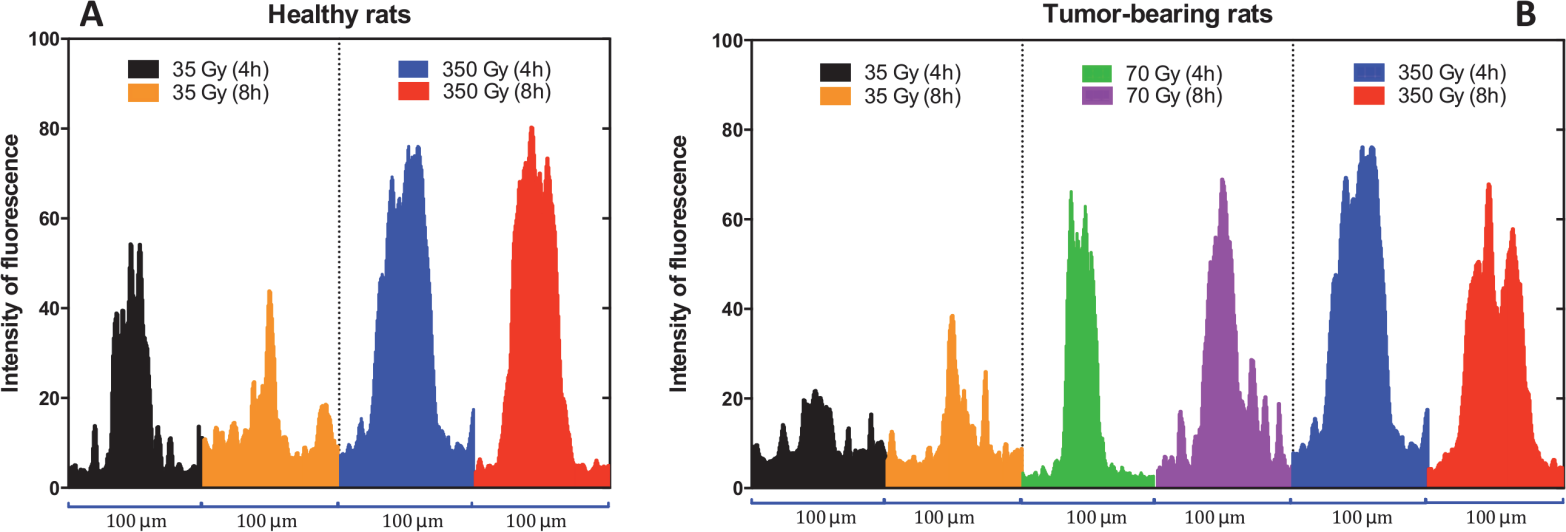
Intensity of the fluorescence measured through a 100 μm profile line traced perpendicular to the direction of the microbeams peak radiation path. A) Intensity profile of normal rats. B) Intensity profile in tumour-bearing animals.

**Fig 6:**
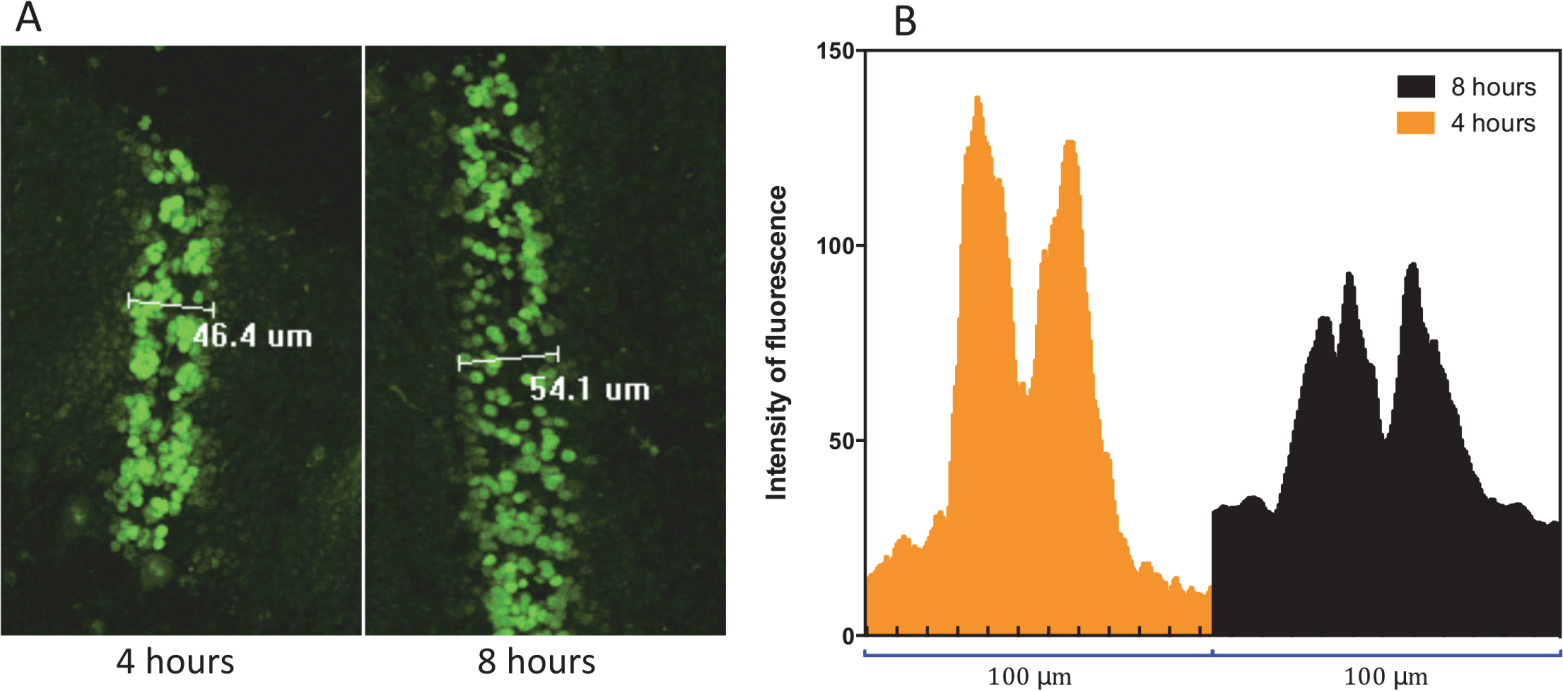
Different width of the radiation paths. A) Microbeam tracks as outlined by γ-H2AX stain at 4 and 8 hours after a 350 Gy irradiation. The high dose delivered resulted in an increase in the width of the microbeam track over time. B) Intensity of the fluorescence of the areas depicted in A.

Fig. 5 demonstrates that the γ-H2AX fluorescence intensity in the cerebellum is directly related to irradiation dose. The histograms in Fig. 5A correspond to those from normal animals indicating that the dose is directly correlated with the intensity of the fluorescence. Additionally, the time interval between irradiation and dissection does not seem to have a major impact on the 350 Gy group, but it shows a clear difference after 35 Gy. Fig. 5B shows that the dose is also correlated with the strength of the fluorescence in tumour-bearing animals. Here, dissection times show clear differences after 35 and 350 Gy irradiation. We attribute these differences to a possible migration of the irradiated cells towards the valley areas, which is explained in the discussion during the analysis of Fig. 6. The presence or absence of a tumour does not seem to have a direct impact on the intensity of the fluorescence.

**Fig 7:**
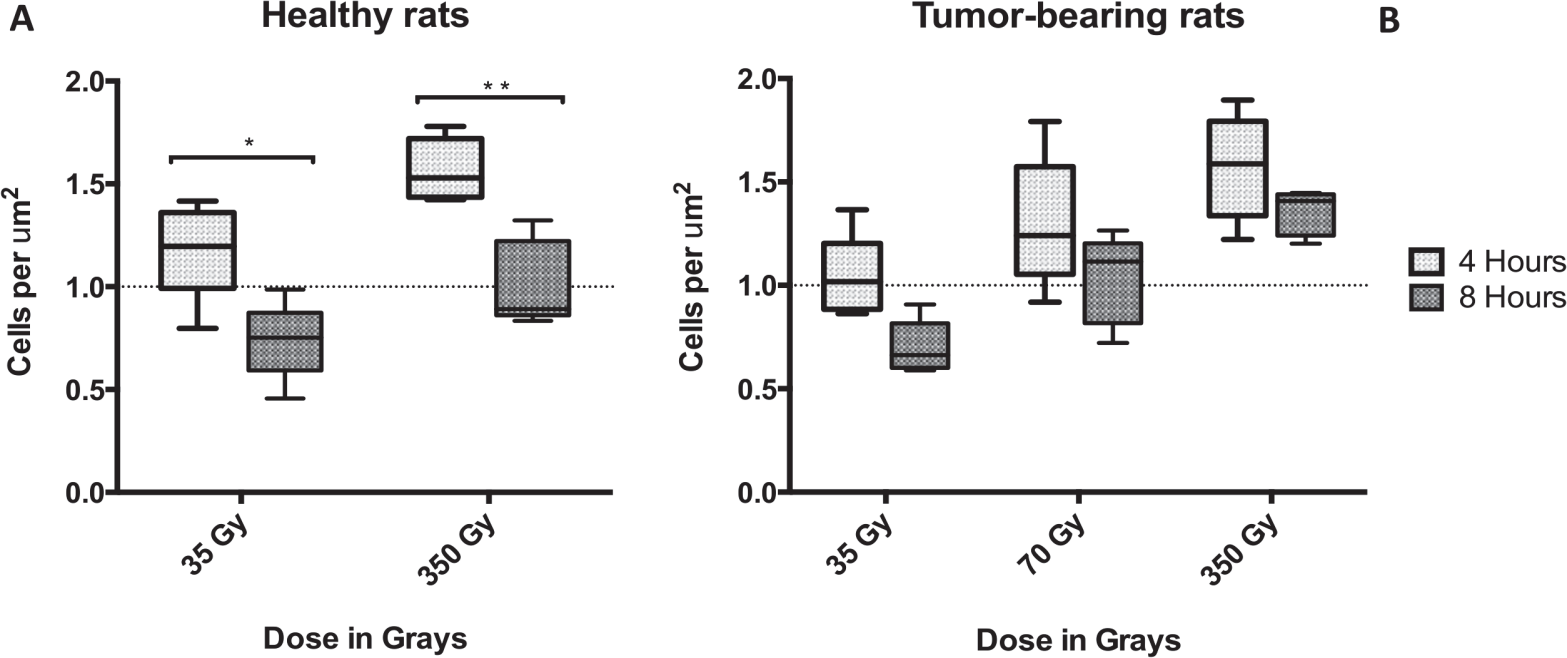
Number of γ-H2AX positive cells per unit area. The measurements were performed in the granular layer of the cerebellum. A) Shows the number of cells in normal rats while B) shows the number of cells in tumour-bearing animals. Stars show significant differences between 4 and 8 hours. Error bars correspond to SD.

The number of γ-H2AX positive cells per micrometer area was assessed and the results are shown in Fig. 7. There was a direct correlation between the number of positive cells per unit area and the radiation dose. The presence or absence of a tumour does not seem to affect this relationship. Although all the groups showed inverse correlation between the number of γ-H2AX positive cells per unit area and the time elapsed after irradiation, this observation was statistically significant (P value < 0.05) only in the normal animals.

## Discussion

The purpose of this work was to study the dose deposition by synchrotron radiation in the brain of Wistar rats using the phosphorylation of the H2AX histone as a biomarker. The conditions explored were 1) different survival times after irradiation to evaluate the dynamics of the γ-H2AX formation over time, 2) different doses of micro- and broad beam synchrotron radiation, and 3) the presence or absence of C6 glioma in the right (irradiated) cerebral hemisphere.

The γ-H2AX antibody stain positively reflected the deposition of the absorbed dose in the brain. The marker clearly outlined the paths of the microbeams and distinguished the irradiated hemisphere from the non-irradiated hemisphere. Our results are in accordance with observations made after synchrotron irradiation of fibroblast monolayers [66] and EMT-6.5 tumours, normal skin, and hair follicle in mouse [7]. The fluorescence observed after the delivery of the broad beam covers a large continuous volume of irradiated tissue in comparison to the much smaller tissue volumes traversed by the microbeams (Fig. 2). The intensity of the fluorescence is stronger in the cerebellum than in the cerebral hemispheres because of the high cellular density of the granular cell layer. It was noted that the irradiation tracks outlined by the y-H2AX biomarker are not always perfectly parallel. This artefact is related to the histology technique. Several authors have described this phenomenon, attributing it to both the process of paraffin embedding and to the distortion of thin tissue sections mounted on glass slides [67,68].

We also studied whether the presence of a tumour could modify the response of brain tissue to synchrotron radiation and lead to a different degree of γ-H2AX formation. Various authors have discussed the phenomenon called tumour-induced bystander effects, which is explained as changes in naïve cells that share the same milieu with cancer cells [69,70]. The tumour microenvironment is established by the interaction between tumour, tumour stroma, the surrounding normal tissues, and the extracellular matrix [71]. This distinctive microenvironment is rich in pro-inflammatory cytokines that contribute to an influx of macrophages and consequent angiogenesis as well as helping the tumour to escape from the immune system [71–75]. Those reasons lead us to hypothesize that the presence of the C6 glioma in the right hemisphere of the brain could modulate the formation of γ-H2AX foci after synchrotron microbeam irradiation through tumour-induced bystander signals. However, our results indicated that there was no significant difference in γ-H2AX intensity, whether animals were normal or harboured a tumour.

When looking at the effect of the dose, our γ-H2AX data indicate that there is a direct correlation between the peak radiation dose and the width of the radiation tracks (Fig. 4), the intensity of the fluorescence (Fig. 5), and the number of immunoreactive cells per unit area (Fig. 7). Our results are in accordance with work published by Rothkamm et al. [55] in which they observed that the intensity and width of the γ-H2AX stripes increased with dose in fibroblasts and the skin of BALB/c mice. Furthermore, microdosimetry calculations published by Spiga et al. [76] also correlated increases in the peak dose with increases in width of the radiation track, which is attributed to the increase in size of the transition zones. Transition or intermediate zones are those where the dose drastically changes from peak to valley levels [27,76]. As the peak dose increases the transition zone increases towards the valley areas, which increases the width of the radiation tracks and may explain our results. In vitro studies developed by Kashino et al. [77] demonstrated that the number of foci per cell was also correlated to the dose in C6 cells. Furthermore, work developed in vivo in subcutaneous tumours and normal skin showed the same correlation between the intensity of γ-H2AX and the dose [7]. The results of this present study further support the data acquired previously by our group [56] in which the brain of nude mice was exposed to synchrotron microbeams and pencil beams.

When evaluating the dynamic of γ-H2AX formation at 4 and 8 hours after irradiation, our results indicated a decrease in the number of γ-H2AX positive cells per unit area over time. This is in accordance with published work in vitro [77] and in vivo [7], where the amount of positive cells per unit area was also assessed after synchrotron microbeam irradiation. Those investigations concluded that the DNA repair mechanisms are the reason of the decrease and our findings shown in Fig. 7 are in agreement with those conclusions. Interestingly, when looking at Fig. 4 in detail, our data show an increase of immunoreactivity over time after 350 Gy, which could be explained by the occurrence of bystander effects within the transition zone and towards the valley area. Although our study shows a connection between bystander effects and synchrotron microbeam radiation therapy, we are not focusing on exploring the nature of the bystander molecule. That would require a larger experiment and the allocation of beam time in the synchrotron is very competitive and thus limited. Instead we focused on demonstrating that bystander effects (no matter the nature of the molecules) can trigger an increase in γ-H2AX signal that is independent of the direct hit by radiation. This idea is supported by work developed from Kashino et al [77] who demonstrated that microplanar beams induced DSBs through bystander effects. Moreover work developed in 3-D tissue models by Sedelnikova et al [78] shows irradiated cells continuously producing DNA-damaging agents which reached a plateau by the first day after alpha-particle microbeam irradiation. Our work shows that the γ-H2AX induction does not only depend on the direct hit by synchrotron radiation, it may as well depend on the actual absolute peak dose triggering different signals in the valley. As previously explained, as the dose increases the transition zone increases towards the tissue immediately adjacent to the nominal width of the microbeam tracks. This ‘transition tissue’ of ≈10–20 μm wide for our MRT spectrum, is therefore exposed to a dose-gradient that may generate a population of cells prone to produce and respond to bystander signals. This would ultimately amplify over time the amount of DNA damage caused by radiation, explaining why the width of the radiation track observed by the γ-H2AX foci increases from 4 to 8 hours. Therefore, our results do not reflect the physical PVDR, instead they suggest that the γ-H2AX should be used to simply observe and describe what is happening after different conditions using the physical dose rather than trying to measure PVDRs.

Although bystander experiments involving the use of microbeams are well described in the literature, a distinction needs to be made between our experiments and those using single versus multiple microbeams, as well as those performed in cell cultures versus animals. The use of a single microbeam is characterized for focusing the radiation to either a single cell or parts of the cells [79–82] or to multiple spots in a small area of cells [83–85]. Those cell culture systems allow for well-controlled experimental settings for the study of mechanisms of transmission of bystander signals and the molecules involved. Our synchrotron work is much more complex because, 1) we use a precise array of multiple rectangular microbeams that aim to target cells within 25μm with very high doses, leaving intermediate gaps of 200μm were cells are also exposed but to a much lower dose level, and 2) we perform our experiments on animals because we are aiming at developing a potential treatment for glioma. Although these differences present obstacles for the study of the bystander effect mechanism, our work provides the first evidence of active involvement of bystander effects in the study and development of innovative methods for brain radiotherapy using synchrotron radiation in vivo.

Fig. 6 shows a comparison of two images 4 and 8 hours after the rats underwent microbeam radiation with a peak dose of 350 Gy. If observed carefully, clusters of cells have increased their relative distance from one another over time. Tofilon and Fike explain that during the first hours after radiation exposure the tissue response is characterized by incipient cell death, and secondary processes—such as bystander effects or inflammation—that generate a persistent oxidative stress environment which will contribute to tissue injury and enhanced cytokine gene expression [86]. Moreover, it is mentioned that those processes are directly related to the absorbed dose. Fig. 6B clearly shows deep decrease in the fluorescence in the center of the profile. The combined analysis of Fig. 6A and 6B suggests that irradiated cells are migrating from the center toward the valley areas. However this claim needs further studies.

In summary, the exploration of γ-H2AX as a biomarker for dose deposition in the brain after synchrotron microbeam radiation brought a number of interesting findings. First, we were able to show a direct correlation between irradiation dose and the formation of γ-H2AX foci in the brain. There was a direct correlation between the width of the radiation track and the dissection time after 350 Gy, while there was no significant change in the width of the microbeam tracks at lower irradiation doses. This suggests that radiation-induced bystander effects are produced from the cells reached by both the high-peak doses and the dose-gradient of the transition zone. The release of these signals would amplify the DNA damage produced by the synchrotron radiation, which would translate in wider γ-H2AX tracks. Secondly, the presence of C6-Glioma does not seem to induce tumour-bystander effects that could modify the degree of γ-H2AX formation. Third, the availability of technical equipment for reliable accurate translation of the targeted animal or human patient is a critical prerequisite for an optimal delivery of synchrotron microbeam radiation. In conclusion, we suggest that the γ-H2AX foci should be used as biomarker for targeted and non-targeted DNA damage after synchrotron radiation rather than a tool to measure the actual physical doses.
